# Prostatic Leiomyoma – Multiparametric Prostate MRI Features

**DOI:** 10.5334/jbsr.1543

**Published:** 2018-04-19

**Authors:** Ortwin Vergauwen, Elke Vereecke, Geert Villeirs

**Affiliations:** 1 Radiology Department, UZ Gent, BE

**Keywords:** Prostate, leiomyoma, MRI

## Case Report

**Figure 1:**
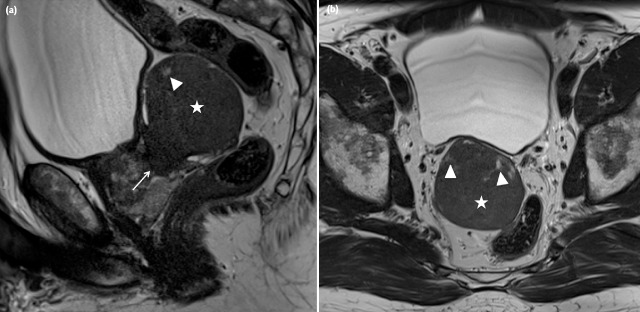
**a)** (sagittal T2w TSE) and **b)** (transverse T2w TSE). T2-weighted imaging showed a sharply demarcated structure (asterisk –5.8 × 6.5 × 5.2 cm) originating from the right part of the transition zone at the level of the midprostate (arrow) with massive posterior bulging The lesion was slightly T2 hyperintense relative to muscle (figure 1a and 1b), with small central T2 hyperintense areas (arrowhead).

**Figure 2:**
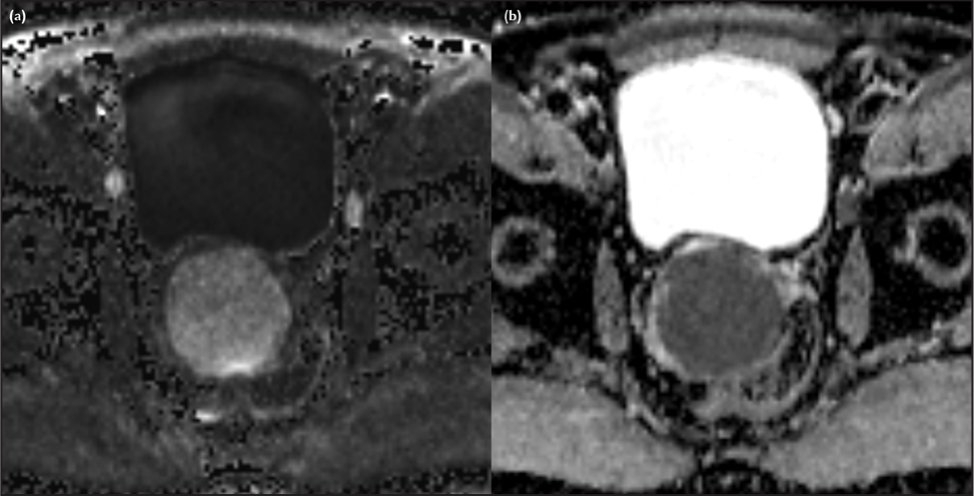
**a)** (b1400) and **b)** (ADC). The lesion has restricted diffusion on transverse high b-value image, corresponding to an ADC of 0.791 ×10^–3^ mm^2^/s.

**Figure 3:**
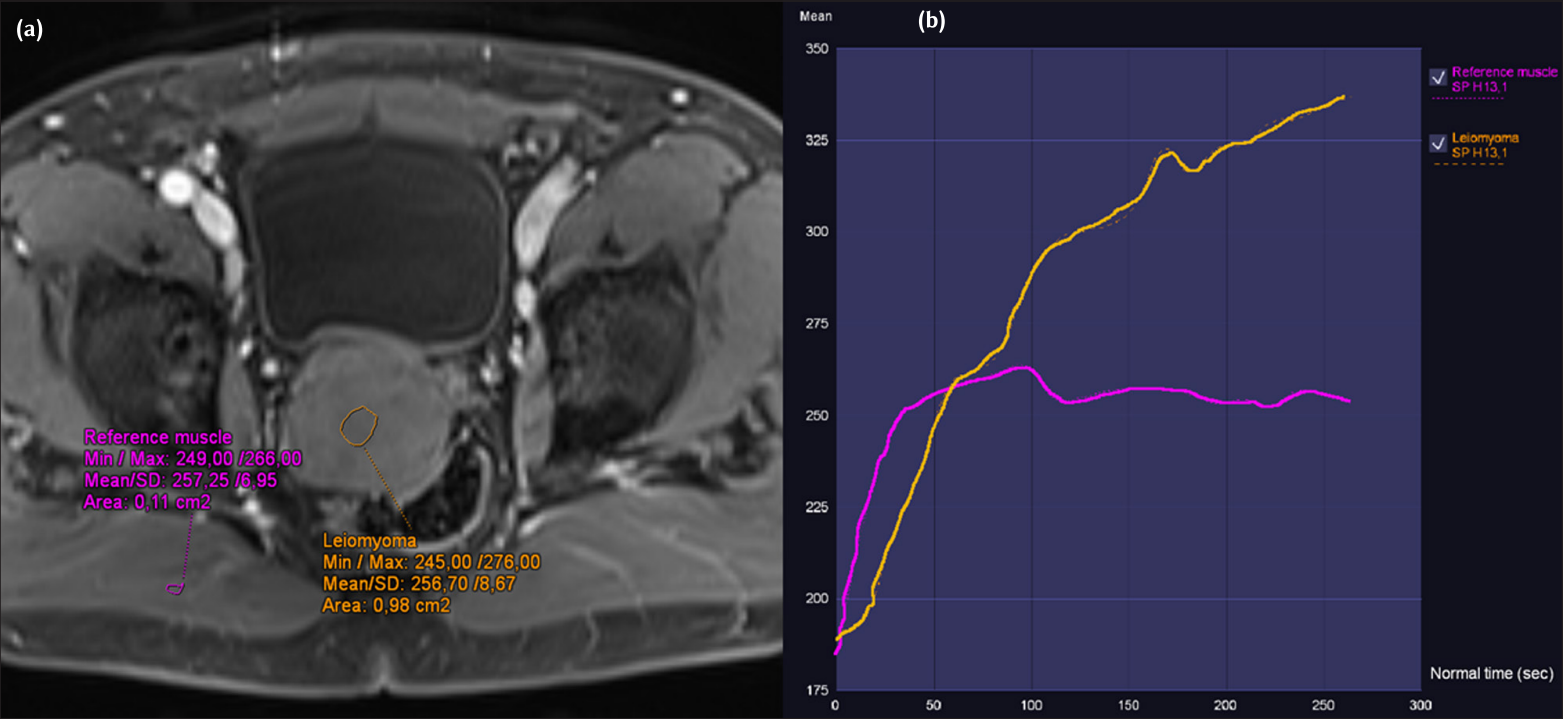
**a)** DCE (75 sec) and **b)** DCE curve. Transverse T1 vibe pre and post intravenous contrast showed homogeneous contrast uptake. The lesion showed slow first pass and progressive enhancement in second pass, the reference muscle (right sides musculus gluteus maximus) showed a slow first pass and a plateau in second pass.

An asymptomatic 62-year-old man presented at the urologist for a check-up with a normal digital rectal examination and a PSA of 1.05 ng/mL. The prostate volume approximated 37 cc on transrectal ultrasound (TRUS) that also showed an hypoechoic structure of 5 cm, extending beyond the prostate, which was interpreted as an utricular cyst. The multiparametric prostate Magnetic Resonance Imaging (MRI) showed a sharply demarcated structure of 5.8 × 6.5 × 5.2 cm (asterisk, Figure 1: a, sagittal, and b, transverse T2w TSE) originating in the right transition zone of the midprostate, with posterior bulging and no invasive behavior. Relative to the muscle, the lesion was overall T1 isointense and slightly T2 hyper-intense, though it contained small T2 hyperintense foci (arrowheads, Figure 1a, b). The lesion had restricted diffusion (Figure 2: a (b1400) and b (ADC)). Dynamic contrast-enhancement showed a homogeneous uptake with a slow first pass and progressive enhancement on second pass (Figure 3: a, transverse post-contrast T1, and b, time-enhancement curve). A benign stromal tumor was suggested. TRUS-guided biopsy was performed, and the tissue diagnosis was a leiomyoma. Considering the absence of clinical complaints and exclusion of malignancy, clinical and imaging follow-up with MRI at six months was advised.

## Discussion

A prostatic leiomyoma is very rare. A leiomyoma occurs in all organs containing smooth muscle cells, but most frequently in the gastrointestinal and female urogenital tract. Within the prostate, it originates from the periglandular tissue, müllerian duct remnants or prostatic capsule. It is an encapsulated mass consisting of smooth muscle cells with various amounts of fibrous tissue, but no glandular elements. The exact etiology is still unknown. Most lesions are large at diagnosis, leading to symptoms of prostatism (decreased force of urination and other obstructive voiding symptoms), as in benign prostatic hyperplasia. Lesions can also be asymptomatic, as in our case. The final diagnosis is made histopathologically and immunohistochemically: it is composed of spindle smooth muscle cells without glandular elements, it expresses smooth muscle actin (SMA) and desmin, and it is negative for CD34, in contrast to a stromal tumor of unknown malignant potential (STUMP).

Imaging findings on ultrasound are nonspecific, showing a mass that can either be hyper- or hypoechoic. CT and MRI show a well-defined pelvic mass originating from the prostate with no invasive features. It can be homogenous or contain areas of cystic degeneration. The non-cystic parts are isodense to muscle on CT, while T1-isointense and T2-hypo- to slightly hyperintense relative to muscle on MRI. Diffusion-weighted imaging shows restricted diffusion with a low ADC value, as in our case with an ADC of 0.791 × 10^–3^ mm^2^/s. DCE has little added value in differentiating the lesion, as it shows enhancement with a nonspecific curve pattern.

Multiparametric prostate MRI can differentiate a leiomyoma from clinically significant prostate cancer. Small leiomyomas can be difficult to differentiate from stromal hyperplastic nodules, but when the lesion is large the differential diagnosis includes STUMP, leiomyoma, or fibrous tumors.

Treatment options include radical prostatectomy, transurethral resection, and more recently, prostate artery embolization. Conservative management with active surveillance is an option for asymptomatic patients.
